# Morphometric Analysis of the Infraorbital Canal, Groove, and Foramen in the Indian Population: A Retrospective Analytical Study

**DOI:** 10.7759/cureus.72367

**Published:** 2024-10-25

**Authors:** Thanzeem Noorul Amin Razak, Lakshmi Rathan A C, Vivek Narayanan, Prashanthi Gurram

**Affiliations:** 1 Department of Oral and Maxillofacial Surgery, SRM Kattankulathur Dental College and Hospital, SRM Institute of Science and Technology (SRMIST), Chengalpattu, IND

**Keywords:** infraorbital canal, infraorbital foramen, infraorbital groove, morphometric analysis, orbit

## Abstract

Aim: The aim of this research is to analyse and assess the anatomical variability of the infraorbital canal, groove, and foramen using high-resolution CT images of the Indian population using mimics software.

Materials and methods: A total of 100 high-resolution CT (HRCT) data (200 hemi-faces) with a range of 18-65 years in Digital Imaging and Communication in Medicine (DICOM) format was incorporated in mimics software. Infraorbital foramen (IOF), infraorbital groove (IOG), and infraorbital canal (IOC) were evaluated retrospectively using seven parameters. Statistical analysis was made, and comparisons were made with respect to gender, side, and age groups.

Results: Out of 100 HRCT data obtained, 74 were of men and 13 were of women, with an average age of 32.5 years. Of 200 hemifaces, the average length of the IOC was 14.99 ± 6.25 mm, the average length of the IOG was 15.25 ± 7.87 mm, the average total length was 30.26 ± 3.36 mm, and the average transverse diameter of IOF was 2.02 ± 0.48 mm. The mean distance from IOF to the infraorbital margin (IOM) was 7.35 ± 1.67 mm, IOC to the medial wall was 16.02 ± 2.22 mm, and IOC to the lateral wall was 16.99 ± 2.47 mm.

Conclusion: Measurement data of infraorbital apparatus varies among different populations. This study contributes valuable knowledge regarding the measurements of the IOC, IOG, and IOF, particularly within the Indian population. This information can help by minimizing the risk of complications and improve the treatment planning for operative and anesthetic procedures.

## Introduction

The orbits are conical structures composed of seven bones and four walls. Even though the medial wall of the orbit is the thinnest bone among these, it is reinforced by the perpendicular septa of the ethmoid sinuses. Second to it is the floor of the orbit, which is unsupported and thin, making it the most vulnerable to fractures during trauma. The orbital floor is formed by the sphenoid, and the orbital processes of both the maxillary bone and palatine bone. The inferior orbital fissure gives rise to the infraorbital groove (IOG), which extends as the infraorbital canal (IOC). The infraorbital nerve (ION) and blood vessels traverse the IOG and IOC, ultimately exiting through the infraorbital foramen (IOF) in the midface region [1].

The ION, a branch of the maxillary nerve, provides sensory innervation to the maxillary teeth and gingiva from the incisor to the premolar region and to the maxillary sinus mucosa. The terminal branches of the ION, which exit through the IOF, supply the skin and mucous membranes of the side of the nose, lower eyelid, upper lip, and midface region.

Orbital floor fractures can occur as part of a zygomaticomaxillary complex fracture or in isolation, referred to as "blowout" fractures. Traditionally, the orbital floor is accessed via the transcutaneous approach or the transconjunctival approach, with the endoscopic approach being another option [2].

Therefore, a precise understanding of the position of the IOF, as well as the morphology and course of the IOC and IOG, is crucial for surgeons. Various studies have utilized human skulls, CT scans with three-dimensional models, and cephalometric analyses to elucidate the morphology and anatomical relationships of these structures [3,4]. While the anatomic variations of the IOF, IOG, and IOC have been analyzed in Indian skulls [5,6], there is a lack of information on the anatomy of the IOF, IOG, and IOC using high-resolution computed tomography (HRCT) in the Indian population.

## Materials and methods

For this retrospective investigation, 100 HRCT images in Digital Imaging and Communication in Medicine (DICOM) format were obtained from the archived records of the Oral and Maxillofacial Surgery Department at our hospital. The institutional ethical committee approved the research investigation (IEC no. SRMIEC-ST0123-736). The sample estimation was done using G-power statistics. With a target power of 80% and a significance level of 0.05, we determined that a total sample size of approximately 100 participants is required.

HRCT files, as well as demographic information, including age and gender, were reviewed from medical records between March 2023 and August 2023, and the data were compiled. Cases with genetic or syndromic conditions, maxillo-mandibular pathological diseases, trauma, skeletal asymmetries or diseases, and images containing artifacts that hinder the identification and measurement of reference points were excluded from this investigation.

All HRCT scans were performed using a G-optima 128-slice machine with a 1 mm slice thickness by a single radiologist specializing in oral and maxillofacial radiology. During acquisition, the patient's head was in a neutral position, and their lips were relaxed while maintaining a bite in centric relation.

**Figure 1:**
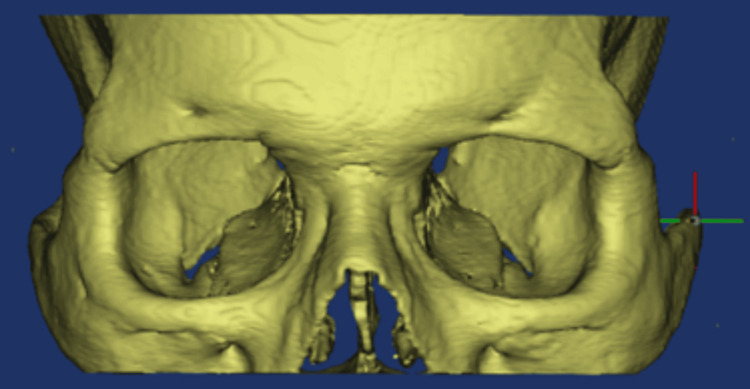
3D model rendered after incorporation of DICOM data into Mimics software. DICOM, Digital Imaging and Communication in Medicine

**Figure 2:**
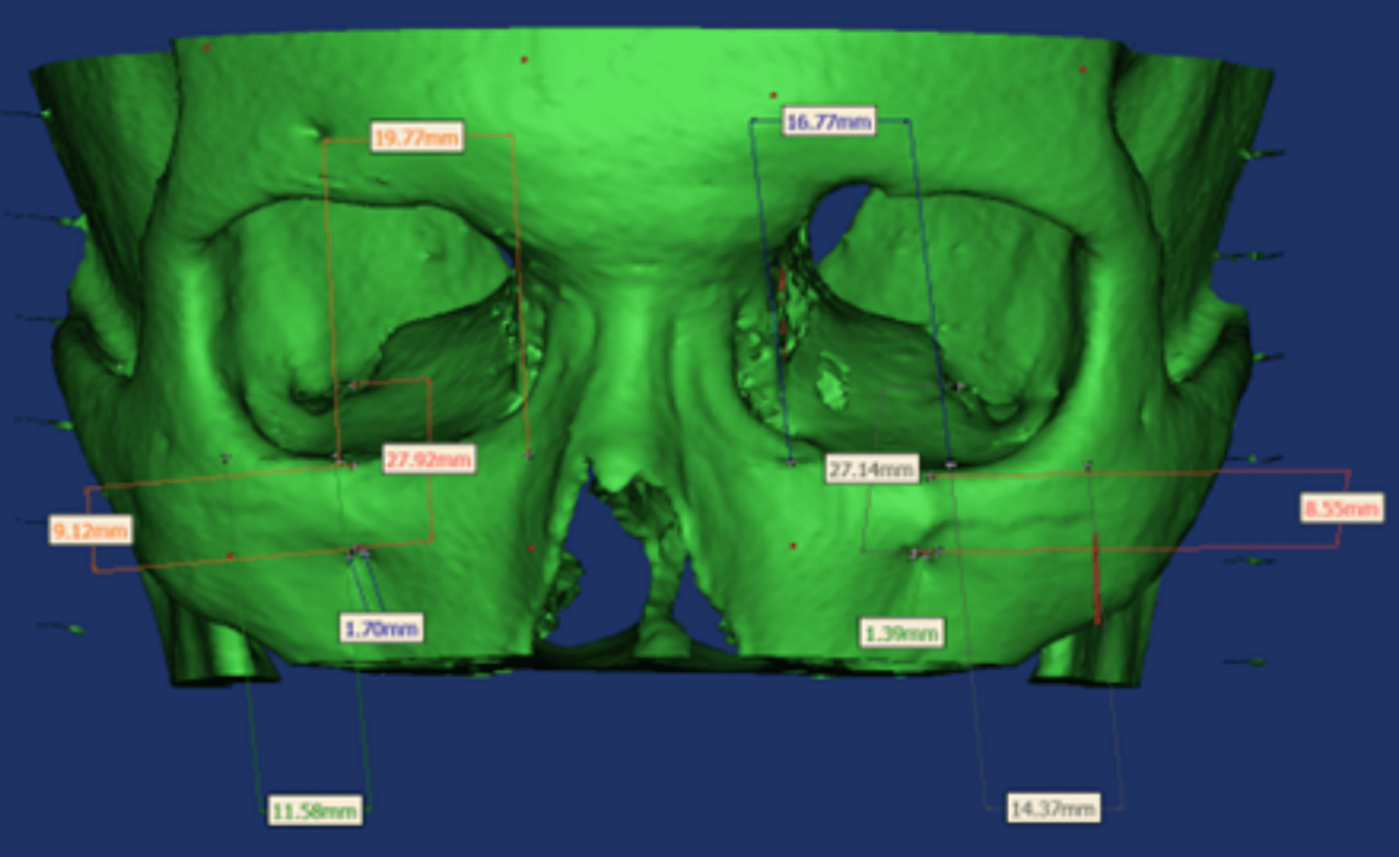
Morphometric analysis using Mimics software.

The DICOM images were imported to Materialise Mimics 19.0 (Materialize NV, Leuven, Belgium) software. A single independent dental intern, well-trained in this software, conducted all morphometric analyses. The measurements were reassessed after a period of one month (Figures 1-2). The evaluated parameters included the following: infraorbital canal length; infraorbital groove length; distance between the infraorbital canal and medial wall; distance between the infraorbital canal and lateral wall; shape of the infraorbital foramen; transverse diameter of the infraorbital foramen; distance between the infraorbital margin (IOM) and the infraorbital foramen; and presence of accessory canals, accessory foramina, and other variations.

Statistical analysis

Statistical Product and Service Solutions (SPSS, version 26.0; IBM SPSS Statistics for Windows, Armonk, NY) was used for analyzing the data with p<0.05 set as the level of significance. To determine the mean and standard deviation of each group, descriptive statistics have been produced. The Shapiro-Wilkinson test has been performed to assess the normality of the data. Inferential statistics to find out the difference between the groups was done using the Mann-Whitney U test or independent t-test for two groups and the Kruskal-Wallis test, followed by the Bonferroni posthoc test for more than two groups.

## Results

Out of 100 HRCT data obtained, 74 were of men and 13 were of women, with an average age of 32.5 years. Of the 200 hemifaces, the average length of the IOC was 14.99 ± 6.25 mm, the average length of the IOG was 15.25 ± 7.87 mm, the average total length was 30.26 ± 3.36 mm, and the average transverse diameter of the IOF was 2.02 ± 0.48 mm. The mean distance from IOF to IOM was 7.35 ± 1.67 mm, IOC to the medial wall was 16.02 ± 2.22 mm, and IOC to the lateral wall was 16.99 ± 2.47 mm (Table 1).

**Table 1:** Descriptive statistics. N, total number of sample

	N	Minimum	Maximum	Mean	Std. Deviation
Infraorbital canal length	200	7.45	29.49	14.9939	6.25296
Infraorbital groove length	200	.00	26.39	15.2543	7.87688
Total length	200	22.32	36.91	30.2691	3.36011
Distance between infraorbital canal and medial wall	200	10.84	19.95	16.0239	2.22468
Distance between infraorbital canal and lateral wall	200	11.58	23.04	16.9990	2.47496
Transverse diameter of infraorbital foramen	200	1.10	3.37	2.0229	.48132
Distance between infraorbital margin and infraorbital foramen	200	3.11	11.64	7.3500	1.67313
Valid N (listwise)	200				

According to our analysis, the length of IOC showed a statistically significant difference between the left and right sides, with a p value of 0.034. The average length of IOC on the right side is 14.25 mm, and that of the left side is 15.73 mm. However, there was no statistically significant difference identified in the length of IOG with regard to sides (Table 2).

**Table 2:** Comparison of length of IOC, IOG, and transverse diameter IOF between right and left sides. A p value of ≤ 0.05 is set as statistically significant. IOG, infraorbital groove, IOC, infraorbital canal, IOF, infraorbital foramen

	Infraorbital canal length	Infraorbital groove length	Total	Transverse diameter of infraorbital foramen
Mann-Whitney U	4134.000	4634.000	4544.000	4768.000
Wilcoxon W	9184.000	9684.000	9594.000	9818.000
Z	-2.116	-0.897	-1.114	-0.567
Asymp. Sig. (2-tailed)	0.034	0.370	0.265	0.571

The distance between IOC and medial wall showed a statistically significant difference between sides with a p value of 0.008. However, no statistically significant difference was present in the distance of IOC to the lateral wall and IOM between the sides (Table 3).

**Table 3:** Comparison of the distance of IOF to medial, lateral, and IOM between the left and right sides. A p value of ≤ 0.05 is set as statistically significant.

	Side	N	Mean	Std. Deviation	Std. Error Mean	P value
Distance between infraorbital foramen and medial wall	Right	100	15.6108	2.20386	0.22039	0.008*
	Left	100	16.4370	2.17867	0.21787	
Distance between infraorbital foramen and lateral wall	Right	100	17.3098	2.43574	0.24357	0.07
	Left	100	16.6882	2.48692	0.24869	
Distance between infraorbital margin and infraorbital foramen	Right	100	7.1678	1.68615	0.16862	0.12
	Left	100	7.5322	1.64828	0.16483	

There is a highly statistically significant difference present in the length of IOC with regard to gender with a p value of 0.0001. The mean length of IOC was 16.04 mm in males and 12.01 mm in females. However, no statistically significant difference was observed in the IOG length with regard to gender (Table 4).

**Table 4:** Comparison of the length of IOC and IOG with respect to gender. A p value of ≤ 0.001 is set as highly statistically significant.

	Infraorbital canal length	Infraorbital groove length	Total	Transverse diameter of infraorbital foramen
Mann-Whitney U	2400.000	3194.000	3764.000	3698.000
Wilcoxon W	3778.000	14220.000	5142.000	14724.000
Z	-4.033	-1.827	-0.234	-0.418
Asymp. Sig. (2-tailed)	0.0001*	0.068	0.815	0.676

A highly statistically significant difference was also identified in the distance of IOF to the medial wall, lateral wall, and infraorbital margin with regard to gender with a p value ≤ 0.001 (Table 5).

**Table 5:** Comparison of the distance of IOF to the medial wall, lateral wall, and IOM with respect to gender. A p value of ≤ 0.001 is set as highly statistically significant.

	Sex	N	Mean	Std. Deviation	Std. Error Mean	P value
Distance between infraorbital foramen and medial wall	Male	148	16.5516	2.08423	0.17132	0.0001*
	Female	52	14.5219	1.91890	0.26610	
Distance between infraorbital foramen and lateral wall	Male	148	17.0120	2.47402	0.20336	0.91
	Female	52	16.9619	2.50142	0.34688	
Distance between infraorbital margin and infraorbital foramen	Male	148	7.6388	1.61787	0.13299	0.0001*
	Female	52	6.5281	1.56448	0.21695	

Among different age categories, the distance between IOF and medial wall and the distance between infraorbital margin and IOF reported significant differences with a p value ≤ 0.05 (Table 6).

**Table 6:** Comparison of the distance of IOF to the medial wall, lateral wall, and IOM with respect to age groups. A p value of ≤ 0.05 is set as statistically significant.

		N	Mean	Std. Deviation	P value
Distance between infraorbital foramen and medial wall	18-34	132	15.6744	2.29732	0.002*
	35-50	48	16.4271	1.90109	
	51-65	20	17.3630	1.83322	
	Total	200	16.0239	2.22468	
Distance between infraorbital foramen and lateral wall	18-34	132	17.2045	2.50113	0.262
	35-50	48	16.6213	2.55918	
	51-65	20	16.5490	1.98908	
	Total	200	16.9990	2.47496	
Distance between infraorbital margin and infraorbital foramen	18-34	132	7.1535	1.81280	0.046*
	35-50	48	7.8129	1.31395	
	51-65	20	7.5360	1.24300	
	Total	200	7.3500	1.67313	

**Figure 3:**
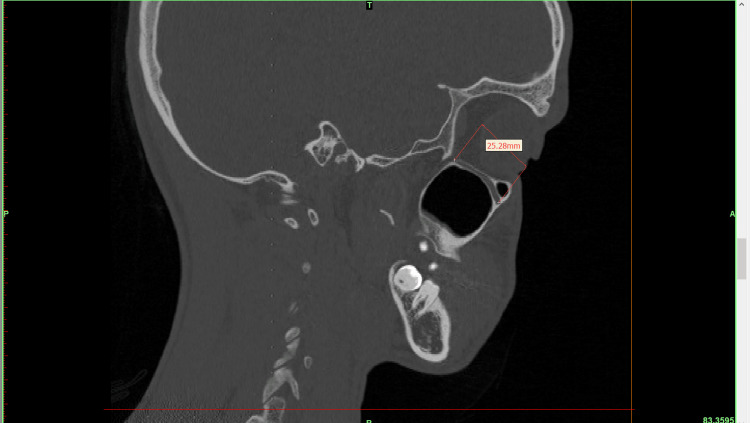
Sagittal section showing the presence of only canal.

The shape of the IOF was triangular in 5% of cases, elliptical in 42%, and oval in 53%. In 18% of cases, only IOC was observed without the presence of IOG (Figure 3), and 3% of cases showed the IOC within the maxillary sinus (Table 7).

**Table 7:** Accessory canals and accessory foramen and other variations. Data represented as %.

Variations		Frequency	Percent	Valid Percent	Cumulative Percent
Valid	Absent	158	79.0	79.0	79.0
	Canal in max sinus	6	3.0	3.0	82.0
	Only canal	36	18.0	18.0	100.0
	Total	200	100.0	100.0	

## Discussion

CT has become a widely utilized imaging modality for the oral and maxillofacial region due to its high contrast resolution, which allows it to distinguish between tissues with physical density differences of less than 1% [7]. This high resolution makes CT particularly advantageous over other radiographic methods. Additionally, three-dimensional reconstruction techniques based on CT provide significant benefits over direct measurements on cadavers or skulls. These methods, facilitated by specialized software, enable the calculation of lengths and angles of numerous anatomical structures and allow for the observation of craniomaxillofacial bones from multiple perspectives [3]. In our study, the dimensions and anatomy of the infraorbital groove, canal, and foramen were measured and analyzed using three-dimensional rendering of HRCT scans.

Orbital blowout fractures frequently occur medial to the IOC on the orbital floor, which can lead to contusion of the ION and deformation of the IOC, resulting in symptomatic dysesthesia in the areas innervated by the ION [8]. In rare cases, chronic pain may occur along the ION distribution [9]. Iatrogenic injuries to the ION can also arise from the treatment of these fractures and other procedures such as tumor surgeries, Caldwell-Luc operations, and Le-Fort osteotomies [10].

Anesthesia of the ION is often employed in various surgical or cosmetic operations involving the soft tissues of the midface region. This includes scar revisions, surgery of maxillary teeth up to the premolars, and management of orbital floor or nasal bone fractures [11,12]. The orientation of the acupuncture point utilized to treat trigeminal neuralgia depends on the position of the IOF [13]. Surgeons must exercise caution when dissecting the periosteum near the IOF during maxillary osteotomies or midfacial lift procedures to treat midfacial ptosis [14]. The recommended local anesthetic technique for early-stage cleft lip surgery and endoscopic endonasal maxillary sinus surgeries is bilateral ION block [15,16]. During surgical procedures, injuries to the infraorbital neurovascular bundle can result in bleeding and numbness in the affected facial regions [17].

As a result, clinicians need to have knowledge of the infraorbital foramen's position, as well as the course and morphology of the Infraorbital groove and canal.

IOC-groove (IOC-G) complex: Accurate assessment of the IOC and IOG lengths in dry skulls can be challenging. Kazkayasi et al. [4] conducted an anatomical and cephalometric study, in which they reported IOC length as 22.95 ± 5.43 mm and IOG length as 5.95 ± 4.90 mm. Hwang et al. [3] measured the IOC and IOG using CT scans and found lengths of 11.7 ± 1.9 mm and 16.7 ± 2.4 mm, respectively. Fontolliet et al. [18] utilized CBCT scans, reporting IOC and IOG lengths of 24.4 ± 2.9 mm and 4.6 ± 1.7 mm, respectively, with a total IOC-G length of 29 ± 3.0 mm. Pryzgocka et al. [19] reported IOC lengths of 14.23 ± 4.68 mm (right) and 13.71 ± 4.62 mm (left) and IOG lengths of 13.49 ± 3.87 mm (right) and 14.14 ± 4.36 mm (left), with a total IOC-G length of 27.71 ± 3.54 mm (right) and 28.11 ± 3.22 mm (left), which is similar to our study. While the total length of the IOC-G complex is similar across studies, marked discrepancies in IOC and IOG lengths arise from variations in the definitions of these structures. Hwang et al. [3] and Bahsi et al. [20] found significant differences in the length of the infraorbital groove between males and females. Our study identified significant differences in IOC length between genders as well as between sides.

IOF diameter and its distance from IOM: Anatomical studies on skulls reported the IOF-IOM distance and transverse diameter of the IOF as 6.16 ± 1.8 mm and 3.35 ± 1.3 mm, respectively, by Singh [6]. Kazkayasi et al. [4] reported a distance of 7.45 ± 0.95 mm between the IOF and IOM. Hwang et al. [3] found an IOF-IOM distance of 9.6 ± 1.7 mm using CT scans. Bahşi et al. [20], using CBCT scans, reported IOF-IOM distances of 7.47 ± 1.40 mm (right) and 7.39 ± 1.41 mm (left) and transverse diameters of 3.37 ± 0.52 mm (right) and 3.22 ± 0.57 mm (left). Fontolliet et al. [18] reported an IOF diameter of 3.0 ± 0.6 mm. In our study on the Indian population, we found an IOF-IOM distance of 7.35 ± 1.67 mm and an IOF diameter of 2.02 ± 0.48 mm. Kara et al. [21] observed significant differences between genders regarding the mean diameter of the IOF, while Apinhasmit et al. [22] noted significant differences in IOF-IOM distance between genders. In our study, we detected significant differences in IOF-IOM distance across different gender and age categories.

Mailleux et al. [23] and Chandra et al. [24] documented cases of IOC traveling through the maxillary sinus. Ference et al. [25] found this pattern in 12.5% of 200 cases, while Bahsi et al. [20] reported a 7% occurrence in 150 cases, and Lantos et al. [26] found it in 10.8% of 500 cases. Our study observed a 3.0% frequency of this variation in 200 hemi-faces and an 18% frequency of hemi-faces having only an IOC without an IOG. M.C. Rusu et al. [27] reported a case where the IOC was replaced by a lateroantral canal on both sides. Hwang et al. [28] analyzed the frequency of accessory IOFs, finding an overall frequency of 16.9% ± 8.6%. Sokhn et al. [29] found accessory foramina in 8.6% of cases in a radiographic study.

The following tabulation shows the comparison of this study with various other studies done in different populations (Table 8).

**Table 8:** Comparison of parameters with the literature. Data represented as mean ± standard deviation. CBCT, Cone beam computed tomography; HRCT, high-resolution CT

Study	Type and Population	IOC length (mm)	IOG length (mm)	IOC-IOG (mm)	IOF-IOM (mm)	IOF diameter (mm)
Hwang et al. [3]	HRCT, Korean	11.7 ± 1.9	16.7 ± 2.4	N/A	9.6 ± 1.7	N/A
Kazkayası et al. [4]	Dry skulls, Turkish	22.95 ± 5.43	5.95 ± 4.90	N/A	7.45 ± 0.95	N/A
Mahajan et al. [5]	Dry skulls, North Indian	N/A	N/A	N/A	5.81	2.6
Singh [6]	Dry skulls, Indian	N/A	N/A	N/A	6.16 ± 1.8	3.35±1.3
Fontolliet et al. [18]	CBCT, Swiss	24.4 ± 2.9	4.6 ± 1.7	29 ± 3.0	N/A	3.0 ± 0.6
Pryzgocka et al. [19]	Dry skulls, Polish	14.23 ± 4.68	13.49 ± 3.87	27.71 ± 3.54	N/A	N/A
Bahşi et al. [20]	CBCT, Turkish	8.28 ± 1.69	22.59 ± 3.88	N/A	7.47 ± 1.40	3.37 ± 0.52
Kara et al. [21]	CT, Turkish	N/A	N/A	N/A	N/A	1.51 ± 0.49
Apinhasmit et al. [22]	Dry skulls, Thai	N/A	N/A	N/A	9.23 ± 2.03	3.35 ± 0.62
Sokhn et al. [29]	Radiograph, Lebanese	N/A	N/A	N/A	7.98 ± 1.41	3.71 ± 0.63
Rahman et al. [30]	Cadaver, American	14	13	N/A	8	N/A
Present study	HRCT, Indian	14.99 ± 6.25	15.25 ± 7.87	30.26 ± 3.36	7.35 ± 1.67	2.02 ± 0.48

This study was performed meticulously to establish reliable identification of infraorbital neurovascular apparatus in the Indian population. However, this study possesses a few limitations, which include small sample size, single-center design, and confined to a particular ethnic/racial group.

## Conclusions

Identifying the properties and variants of the IOC, IOG, and IOF is crucial for preventing infraorbital nerve damage and minimizing complications during surgeries involving the orbital floor, as well as other procedures such as anesthesia. Measurement data for these anatomical structures vary among different populations and across different studies. This study contributes valuable knowledge regarding the measurements of the IOC, IOG, and IOF, particularly within the Indian population. This information can help minimize the risk of complications and improve the treatment planning for operative and anesthetic procedures.
